# The contribution of the CTRP family and its receptors to fibrotic diseases

**DOI:** 10.3389/fimmu.2026.1869146

**Published:** 2026-10-19

**Authors:** Shiena Tanaka, Hina Nozawai, Hsi-Hua Chi, Hiroki Ueno, Yayoi Tsuruzoe, Nanami Sakaguchi, Rio Tsukamoto, Shoko Fujiyoshi, Sung-Il Lee, Masanori A. Murayama

**Affiliations:** Department of Animal Models for Human Diseases, Institute of Biomedical Science, Kansai Medical University, Osaka, Japan

**Keywords:** AdipoR, C1q/TNF, CTRP, fibrosis, PAQR

## Abstract

Adiponectin and other 15 members of the complement C1q/tumor necrosis factor (TNF)-related protein (CTRP) are proteins secreted primarily from adipose tissue; furthermore, recent studies have revealed their expression in various other tissues. Beyond their roles as adipokines, CTRPs critically regulate fibrogenic responses in renal and cardiovascular tissues. Adiponectin exerts its biological functions via AdipoR1, AdipoR2, and Cadherin-13 (known as T-cadherin), while several CTRPs share AdipoR1 and AdipoR2 as functional receptors. This review summarizes the current understanding of the protective effects of the CTRP family in fibrotic diseases. Future studies utilizing gain-of-function and loss-of-function animal models with single or double genetic mutations will elucidate the detailed molecular mechanisms, including the identification of functional receptors, involved in CTRP-mediated regulation of fibrotic diseases.

## 1 Introduction

C1q is a key component of the classical pathway in complement activation. The binding of C1q to immune complexes via the ligand-recognition C-terminal globular C1q domain induces a conformational change leading to the autoactivation of other complement components (1). Various proteins, including non-complement proteins, also possess the globular C1q signature domain. The most extensively and thoroughly investigated representative of globular C1q-possessing proteins is adiponectin (also known as ACRP30 or AdipoQ). Adipocytes produce Adiponectin, a major adipokine that acts as a hormone (2). Adiponectin is a key regulator of metabolic homeostasis; consequently, Adiponectin deficiency or resistance contributes to various metabolic syndromes, such as type 2 diabetes (3, 4). Due to the structural similarities between the globular C1q domain of Adiponectin and globular domain tumor necrosis factor (TNF) superfamily, including folding topology, key residue conservations, trimer interfaces, and intron positions, researchers suggested an evolutionary link that underlies the C1q/TNF superfamily (5). Subsequently, researchers designated Adiponectin and its paralogs as C1q/TNF-related proteins (CTRPs) (6).

CTRPs consist of four regions including an N-terminal signal sequence, a short variable region, a collagenous domain, and a C-terminal complement C1q-like globular domain. After synthesis, CTRPs undergo post-translational modifications, such as glycosylation and hydroxylation. Through these post-translational modification, Adiponectin forms three oligomeric isoforms: the low-molecular-weight (LMW, trimer), middle-molecular-weight (MMW, hexamer), and high-molecular-weight (HMW, multimer) forms (7). Multimerization depends on several conserved amino acids in the collagen domain; these post-translational modifications drive the formation of hexameric and HMW forms of Adiponectin (8, 9). Adiponectin circulates in the plasma in both full-length and globular forms; the latter results from proteolytic cleavage (10). Researchers have identified AdipoR1, AdipoR2, and Cadherin-13 (known as T-cadherin) as functional Adiponectin receptors. Both AdipoR1 and AdipoR2 bind the trimeric LMW and globular forms of Adiponectin, although their binding affinities for these ligands differ (11). Cadherin-13 binds the MMW and HMW forms of Adiponectin (12). Furthermore, PAQR3 (also known as AdipoR3; RKTG) is the candidate fourth receptor for Adiponectin and localize to the Golgi apparatus (13). Interestingly, AdipoR1 and AdipoR2 form homomers and heteromers, exhibiting distinct ligand binding and signaling properties (14).

In the following sections, we briefly summarize the circumstances of the discovery and receptor information for other CTRPs (**Table 1**). CTRP1 (also known as G protein-coupled receptor-interacting protein, GIP) acts as an inhibitor of collagen-induced platelet aggregation by competitively binding to von Willebrand factor (15). Although the functional receptor for CTRP1 remains unidentified, relaxin family peptide receptor 1 (RXFP1) is a potential candidate (16). Like other CTRPs, CTRP2 improves insulin and lipid tolerance in diabetes via unknown receptors (17). CTRP3 (also known as CORS26, Cartonectin, or Cartducin) acts as a growth factor for chondrocytes (18), and this biological function is mediated via AdipoR2 (19). Furthermore, ligand-receptor capture technology has revealed that CTRP3 binds to lysosomal-associated membrane protein 1 (LAMP1) (20). CTRP4 is a unique member with two tandem C1q globular domains; adipose tissue and central nervous system highly express this protein (21). In Th17 cells, CTRP4 can competitively bind IL-6R (22). In contrast, CTRP3 inhibits Th17 cell differentiation via AdipoR2 (23). CTRP5 is known as a risk factor for late-onset retinal degeneration (L-ORD). A missense mutation in the CTRP5 gene causes retinal degeneration. Immunofluorescence microscopy analysis and co-immunoprecipitation assays show that CTRP5 interacts with AdipoR1, but not with AdipoR2 (24). Interestingly, depletion of AdipoR1 and AdipoR2 does not affect the metabolic effects of CTRP5 (25). Thus, the functional CTRP5 receptors remain unclear. CTRP6 acts as a complement regulator (26). In addition, CTRP6 regulates adipocyte proliferation and differentiation via AdipoR1 (27). Furthermore, RXFP1 is a candidate receptor for CTRP6 (16). CTRP7 is a biomarker for metabolic syndrome (28). RNA interference experiments suggest that AdipoR1, but not AdipoR2, is the receptor for CTRP7 on cardiocytes (29). CTRP8 contributes to brain cancer development (30) and is the first CTRP identified as a ligand for RXFP1 (16). CTRP9 is an adipokine that forms heterotrimers with Adiponectin (31). Currently, it is well known that CTRP9 interacts with AdipoR1 as a functional receptor (32). CTRP12 (also known as Adipolin, C1qDC2) is an insulin-sensitizing adipokine (33) that exerts biological function via AdipoR2 (34). Erythroferrone, which is designated as CTRP15 (also known as Myonectin) is a myokine (35) and an erythroid regulator of iron metabolism (36). The CTRP15 receptor remains unidentified.

**Table 1:** Summary of CTRPs, and its receptors.

Name	Gene symbol	Other major names	Heterotrimerization with	Interactions (including putative receptors)	Fibrotic response		
					Kidney	Heart	Liver
Adiponectin	*ADIPOQ*	ACRP30, AdipoQ	CTRP2, CTRP9	AdipoR1, AdipoR2, PAQR3, Cadherin-13(CDH13)	↓↓	↓↓	↓↓
CTRP1	*C1QTNF1*	GIP	CTRP6, CTRP14	AdipoR1(?)*, RXFP1(?)	↓↓	±	??
CTRP2	*C1QTNF2*	–	Adiponectin, CTRP7, CTRP15	Unknown	??	??	??
CTRP3	*C1QTNF3*	CORS26, Cartonectin, Cartducin	–	AdipoR2, LAMP1	↓↓	↓↓	↓↓
CTRP4	*C1QTNF4*	–	–	IL-6R (competitive binding)	??	??	??
CTRP5	*C1QTNF5*	–	CTRP15	AdipoR1(?)	??	±	↑↑
CTRP6	*C1QTNF6*	–	CTRP1	AdipoR1, RXFP1(?)	↓↓	↓↓	??
CTRP7	*C1QTNF7*	–	CTRP2	AdipoR1(?)**	??	↓↓	↑↑
CTRP8	*C1QTNF8*	–	CTRP9, CTRP14	RXFP1	??	??	??
CTRP9	*C1QTNF9*	–	Adiponectin, CTRP8, CTRP14	AdipoR1	↓↓	↓↓	??
CTRP10	*C1QL2*	C1qL2	CTRP13, CTRP14, CTRP15	ADGRB3, Neurexin-3, GluK2, GluK4	??	??	??
CTRP11	*C1QL4*	C1qL4	–	ADGRB3	??	??	??
CTRP12	*C1QTNF12*	Adipolin, C1qDC2	CTRP15	AdipoR2	↓↓	↓↓	??
CTRP13	*C1QL3*	C1qL3	CTRP10	ADGRB3, Neurexin-3, NPTX1, NPTXR, GluK2, GluK4	↓↓	↓↓	↓↓
CTRP14	*C1QL1*	C1qL1, CRF	CTRP1, CTRP8, CTRP9, CTRP10	ADGRB3, Neurexin-3	??	??	??
CTRP15	*ERFE*	Erythroferrone, ERFE, Myonectin	CTRP2, CTRP5, CTRP10, CTRP12	Unknown	??	↓↓	??

This table summarizes the alternative names, reported heterotrimerization partners, identified or putative receptors, and the reported effects of CTRPs on fibrosis in the kidney, cardiovascular system, and liver. Receptors followed by “(?)” represent putative receptors that require further experimental validation. *CTRP1-AdipoR1 reflects a macrophage-specific, pro-fibrotic pathway in cardiac tissue, distinct from the RXFP1-mediated protective pathway. **CTRP7-AdipoR1 is based on RNA interference in cardiomyoblasts only and is marked putative in all tissues. Downward arrows (↓↓) indicate anti-fibrotic effects, upward arrows (↑↑) indicate pro-fibrotic effects, “±” indicates inconsistent or context-dependent findings, and “??” denotes that sufficient evidence is currently unavailable. Importantly, anti- or pro-fibrotic effects are assigned only when fibrosis-related endpoints are directly evaluated, such as extracellular matrix or collagen deposition, fibrotic area, established fibrosis-related markers, fibroblast activation, or myofibroblast differentiation; effects on other cardiovascular processes, such as angiogenesis or atherosclerotic plaque formation, are not considered direct evidence of effects on fibrosis.

C1QL1, C1QL2, C1QL3, and C1QL4 are also designated as CTRP14 (also known as C1q-related factor, CRF), CTRP10, CTRP13, and CTRP11, respectively. Initial studies demonstrated that C1QL proteins are secreted molecules with distinct expression patterns in the central nervous system and the ability to form homo- and hetero-oligomeric complexes (37, 38). Subsequently, C1QL proteins were identified as ligands for the adhesion-type G-protein-coupled receptor B3 (ADGRB3), which is also known as brain-specific angiogenesis inhibitor 3 (BAI3), suggesting a role in synapse organization (39). This concept was further strengthened by the demonstration that CTRP14 forms a trans-synaptic complex with presynaptic neurexin-3 and postsynaptic BAI3, thereby regulating synapse formation and maintenance (40). In addition, CTRP10 and CTRP13 were shown to interact with neurexin-3 through the sequence encoded by neurexin-3 exon 25b and to bind the amino-terminal domain of kainate receptors (GluK2, GluK4), suggesting diverse roles of C1QL proteins in synaptic organization and signaling (41). In contrast, although CTRP11 can bind BAI3 *in vitro*, it is predominantly expressed in adipose tissue and testis, and no definitive *in vivo* role in synapse organization has been demonstrated (42). Recent reviews have summarized these functional differences and the diverse physiological roles of the C1QL family (43).

Complement C1q is a multimeric protein composed of three homologous distinct proteins; C1qA, C1qB, and C1qC (44). In turn, CTRPs form both homomeric and heteromeric complexes, such as Adiponectin/CTRP2, Adiponectin/CTRP9, CTRP1/CTRP6, CTRP1/CTRP14, CTRP2/CTRP7, CTRP2/CTRP15, CTRP5/CTRP15, CTRP8/CTRP9, CTRP8/CTRP14, CTRP9/CTRP14, CTRP10/CTRP13, CTRP10/CTRP14, CTRP10/CTRP15, and CTRP12/CTRP15 (35, 45–47) (**Table 1**). C1qA, C1qB, and C1qC in C1q complex have different ligand specificity (48). Interestingly, AdipoR1 and AdipoR2 form homomers and also heteromers. Signal dependency is difference on AdipoR1 homomer, AdipoR2 homomer, and AdipoR1/R2 heteromer (14). Thus, heteromeric CTRPs may provide a mechanism to potentially generate functionally distinct ligands.

Similar to adipokine, many CTRPs regulate metabolic homeostasis (49). Furthermore, CTRP family members contribute to the regulation of fibrotic diseases in various aspects. In this review, we discuss how CTRP receptor selectivity, heteromerization, and immune regulation may collectively influence fibrotic responses across different tissues. Importantly, the evidence supporting CTRP–receptor relationships varies substantially among CTRP family members. In this review, we distinguish three levels of evidence: [1] a demonstrated functional receptor, supported by functional ligand–receptor interaction in the relevant tissue or cell type; [2[an experimentally identified binding partner, demonstrated by *in vitro* binding or interaction assays but without evidence that the interaction mediates the biological effect in the tissue of interest; and [3] a candidate receptor inferred from receptor expression or indirect experimental evidence. These categories should not be considered equivalent, and we use this distinction throughout the text, tables, and figures.

## 2 The role of the CTRP family in the fibrotic response of renal diseases

Renal fibrosis is a major prognostic feature of irreversible renal impairment in chronic kidney disease (CKD) (50). Studies have demonstrated a strong association between serum Adiponectin levels and the outcomes of patients with CKD (51). Adiponectin attenuates renal fibrosis and injury in CKD mouse models (52, 53) and improves various phenotypes in diabetic nephropathy mouse models (54, 55) by activating multiple protective pathways (56, 57).

As with Adiponectin (58), CTRP6 inhibits cell proliferation and extracellular matrix (ECM) expression in mesangial cells (59), and attenuates renal I/R injury *in vivo* (60). Circulating CTRP9 levels decrease in diabetic db/db mice. Replenishing CTRP9 ameliorates renal dysfunction and injury by inhibiting glomerular and tubular glycogen accumulation, fibrosis, oxidative stress, and apoptosis in diabetic nephropathy mice (61). Adiponectin, CTRP6, and CTRP9 share AdipoR1 as a functional receptor (27, 32). AdipoR1 is expressed in the cells constituting the glomerulus: endothelial cells, podocytes, mesangial cells, and Bowman’s capsule epithelial cells, as well as in proximal tubular cells (62, 63). AdipoR1 deficiency causes profibrotic ECM remodeling (64); conversely, this receptor promotes the repair of renal tubular epithelial cells (TECs) (65, 66) and suppresses inflammatory responses in these cells (67). CTRP6 has been reported to interact with AdipoR1 in porcine adipocytes; however, whether this interaction represents a functional receptor axis in renal fibrosis remains to be experimentally validated. Thus, Adiponectin, CTRP6, and CTRP9 may contribute to the regulation of renal fibrosis and other renal functions via AdipoR1.

Renal CTRP3 expression decreases in patients with severe CKD. Furthermore, CTRP3 alleviates renal fibrosis in a CKD rat model by regulating the fibrotic response of human renal proximal TECs (68). In murine cellular models, CTRP3 exerts biological functions via AdipoR2 in chondrogenesis and Th17 cell differentiation (19, 23). Similar to CTRP3 (69), CTRP12 attenuates renal injury by reducing renal inflammation in human TECs via AdipoR2 (34). Adiponectin, CTRP3, and CTRP12 share AdipoR2 as a functional receptor, and this receptor is expressed to a lesser degree on mouse podocytes and human proximal TECs (34, 62, 63). Thus, Adiponectin, CTRP3, and CTRP12 may contribute to the regulation of renal fibrosis in TECs via AdipoR2.

CTRP1 attenuates renal fibrosis via AMPK signaling in a renal injury mouse model (70), whereas CTRP1 deficiency causes renal hypertrophy and dysregulation in mice (71). CTRP1 also attenuates kidney inflammation and fibrosis by regulating macrophage activation in a mouse model (72). Importantly, identifying the exact functional receptors for CTRP1 remains a critical challenge in ligand-receptor biology. In a published textual statement within their discussion section, Peterson et al. (73) noted that the overexpression of AdipoR1, AdipoR2, or Cadherin-13 (known as T-cadherin) failed to enhance recombinant CTRP1 binding. However, it is worth noting that no standalone data figures validating this specific negative interaction were presented in their study, and the precise cellular or species origin of that pilot binding assay was left undisclosed. According to human cell model results, the CTRP8 receptor, RXFP1, has emerged as a potential candidate for CTRP1 and CTRP6 receptors (16). RXFP1 contributes to the inhibition of renal myofibroblast differentiation in mouse and rat cell models (74). Hence, RXFP1 emerges as a promising candidate receptor mediating CTRP1-induced renal protection, although further investigation is warranted to definitively validate this putative axis *in vivo*. Serum CTRP13 levels decrease in renal injury model mice. CTRP13 treatment inhibits ECM accumulation by regulating TGF-β/Smad3 signaling and attenuates inflammation by modulating NF-κB signaling in the injured kidneys in mice (75). C1qL proteins bind ADGRB3 (also known as BAI3) (38). This assignment draws on a comprehensive review of adhesion GPCRs in the kidney (76), which in turn compiles this specific observation from the Nephroseq database, a transcriptomic (microarray-based) data-mining resource; the underlying evidence is therefore mRNA-level enrichment across the whole glomerulus in adult human kidneys. Thus, the CTRP13/ADGRB3 (BAI3) axis should be regarded as a speculative association with renal fibrosis.

The conversion of renal erythropoietin-producing cells to myofibroblasts causes anemia and fibrosis in mouse CKD models (77). Although the contribution of CTRP15 to renal fibrosis remains unclear, CTRP15 deficiency does not significantly affect the improvement of CKD-associated anemia mediated by a hypoxia-inducible factor prolyl hydroxylase (HIF-PH) inhibitor in mice (78). In contrast, erythroid-specific overexpression of CTRP15 ameliorates anemia by improving systemic oxygenation without affecting renal injury markers in adenine-induced CKD mice (79).

These reports show that CTRPs play an important role in regulating renal fibrosis in CKD via AdipoRs and other receptors (**Figure 1A**). AdipoRon, an AdipoR1/AdipoR2 agonist, was developed as a CTRP-targeted agent. Hypertension-induced epithelial-mesenchymal transition (EMT) is a major mechanism of renal fibrosis. AdipoRon attenuates EMT and renal fibrosis in hypertensive mouse models (80) and ameliorates intrarenal fibrosis and other features of diabetic nephropathy in type 2 diabetes mouse models (81). Thus, the CTRPs/AdipoRs axes represent prospective therapeutic targets for renal fibrosis-related diseases.

**Figure 1:**
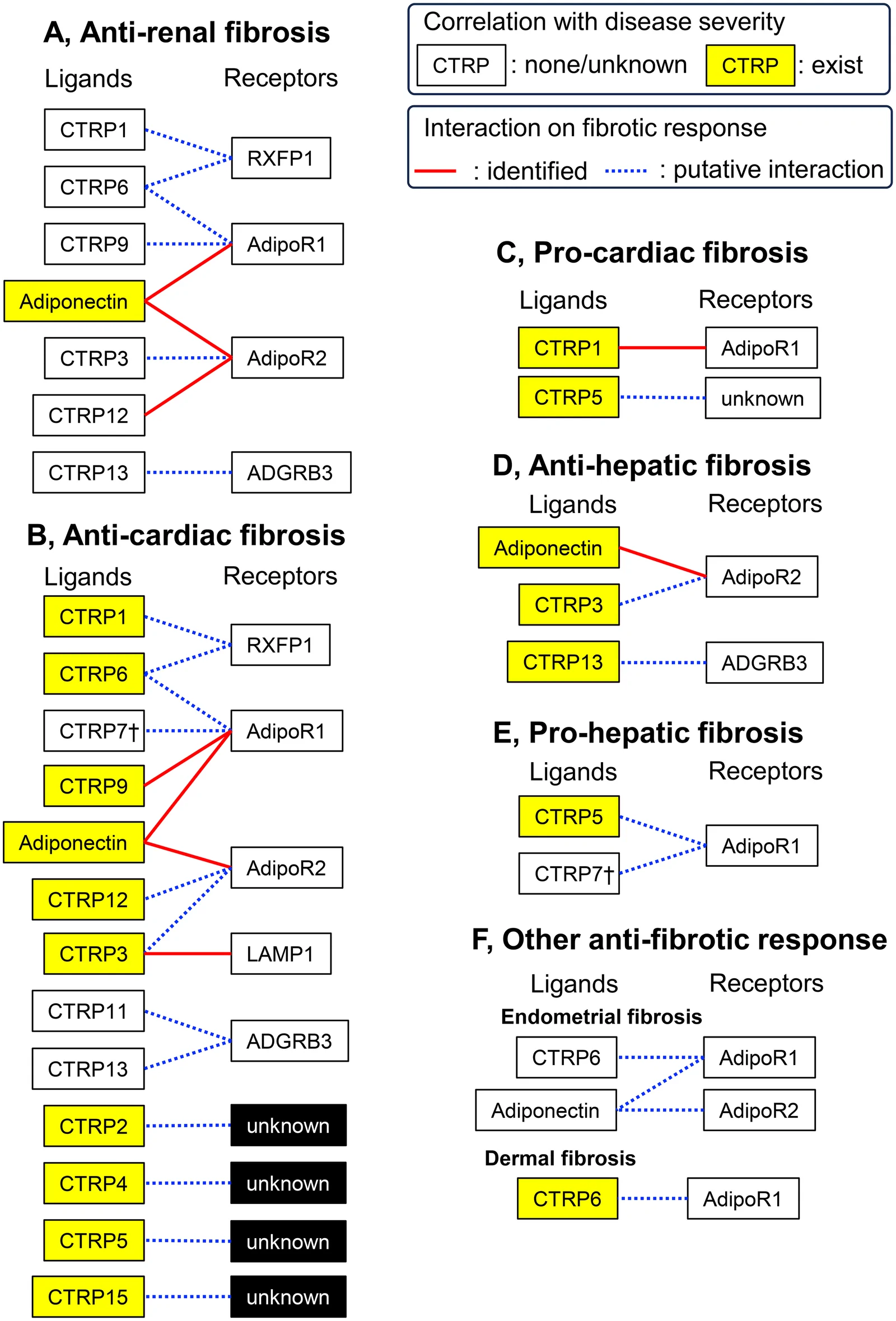
The interaction of CTRPs and its receptors in the regulation of fibrotic response. This schematic summarizes the reported functions of individual CTRPs across major fibrotic diseases, including renal **(A)**, cardiovascular **(B, C)**, hepatic **(D, E)**, and other fibrosis **(F)**. The figure highlights whether each CTRP predominantly exerts protective [anti-fibrotic, **(A, B, D, F)**] or pathogenic [pro-fibrotic, **(C, E)**] effects with identified or putative receptors based on current experimental evidence. Solid red lines indicate identified CTRP ligand-receptor interaction, and dotted blue lines indicate putative CTRP ligand-receptor interaction. The plasma or serum levels of CTRPs associated with severity of patients with fibrotic diseases are yellow-highlighted. †CTRP7-AdipoR1: RNAi evidence in cardiomyoblasts only.

An open mechanistic question raised by this pattern is whether receptor selectivity — AdipoR1 for adiponectin, CTRP6, and CTRP9; AdipoR2 for adiponectin, CTRP3, and CTRP12; RXFP1 for CTRP1; and the candidate ADGRB3(BAI3) axis for CTRP13 — explains why so many structurally related CTRPs converge on the same anti-fibrotic, ECM-limiting outcome in the kidney, or whether this instead reflects redundant, receptor-independent convergence on a shared downstream pathway. Although these studies consistently support the protective roles of CTRPs in renal fibrosis, most evidence is derived from rodent models, and validation in human tissues is required. From a critical perspective, clinical cohort data remain limited, and an analysis of the correlation between circulating CTRPs levels and disease severity is required. Collectively, despite differences among individual CTRPs, most anti-fibrotic CTRPs converge on the regulation of ECM production. The shared mechanism suggests that CTRPs regulate renal fibrosis through coordinated control rather than completely independent pathways. A further open question is what heteromerization contributes to this picture: CTRP12 and CTRP15, both discussed above for their respective roles in renal fibrosis and CKD-associated anemia, have been reported to form a CTRP12/CTRP15 heterodimer, and it remains unknown whether this heteromeric form has a distinct receptor preference or renal activity from either monomeric ligand. To understand the biological mechanisms of these CTRPs via receptors such as AdipoR1 and AdipoR2 on renal fibrosis, genetic loss-of function of putative receptors approaches is necessary.

## 3 Roles of the CTRP family in the fibrotic responses in coronary artery disease and cardiovascular disease

Excessive collagen buildup leads to cardiac fibrosis, contributing to coronary artery disease (CAD) and cardiovascular disease (CVD), including myocardial infarction and hypertension (82). Adiponectin, CTRP1, CTRP3, CTRP5, CTRP6, CTRP7, CTRP9, CTRP12, CTRP13, and CTRP15 may influence CAD and CVD progression through effects on cardiac fibrosis as well as other cardiovascular processes, according to clinical and experimental studies (70, 83, 84). Experimental studies have shown that several CTRPs regulate cardiac fibrosis whereas other affect cardiovascular processes that are not directly related to fibrosis (**Figures 1B, C**).

Serum Adiponectin levels correlate with subclinical myocardial fibrosis in patients with HIV (85). Adiponectin deficiency induces cardiac ECM remodeling, whereas Adiponectin replenishment attenuates this process following cardiac injury in mice (86, 87). Specifically, Adiponectin protects against TGF-β/Smad3- and Arginase II (ArgII)-induced cardiac fibrosis in rodent models (88–90), as well as oxidative stress-induced mouse and rat cardiac myocyte remodeling (91).

Serum CTRP6 levels reportedly increase in patients with CAD (92), but another study showed that serum CTRP6 levels are lower in patients with CAD than in controls (93). CTRP6 levels show sex differences in controls but not in patients with CAD and are associated with BMI in controls and patients with CAD (92). In fact, CTRP6 levels are increased in patients with CAD in the presence of type 2 diabetes (CAD + T2DM) than patients with CAD in absence of type 2 diabetes; furthermore, there is no significant statistical difference between CAD + T2DM and non-CAD + T2DM patients (93). CTRP6 is positively associated with the risk of CAD (92); however, BMI, other metabolic factors, and sex differences are important for the expression of CTRP6. In heart failure model mice, CTRP6 suppresses the differentiation of cardiac fibroblasts to myofibroblasts via the AMPK pathway, and CTRP6 replenishment ameliorates cardiac dysfunction in heart failure model mice (94–96). CTRP6 exerts protective effects against myocardial ischemia/reperfusion (I/R) injury in a rat model (97). CTRP6 also inhibits the proliferation and migration of vascular smooth muscle cells, which play critical roles in atherosclerosis development (98). In spontaneously hypertensive rats, CTRP6 improves ArgII-induced hypertension and vascular endothelial dysfunction by suppressing apoptosis in vascular endothelial cells (99). Plasma CTRP9 levels correlate with disease severity and prognosis in patients with CAD (100). Furthermore, researchers have identified a CTRP9 polymorphism associated in CAD (101). Cardiomyocyte CTRP9 expression decreases during chronic intermittent hypoxia-exacerbated post-myocardial infarction heart failure; subsequently, CTRP9 replenishment attenuates cardiac remodeling in mice (102). Furthermore, CTRP9 deficiency aggravates high-fat diet-induced myocardial hypertrophy and fibrosis by increasing endoplasmic reticulum (ER) stress-related apoptosis in the heart in a mouse model, and CTRP9 replenishment rescues this phenotype by alleviating cardiac lipotoxicity via the LKB1/AMPK pathway (103). Cardiac CTRP9 also contributes to the inhibition of myocardial fibrosis by regulating autophagy in a diabetic cardiomyopathy mouse model (104). CTRP9 overexpression attenuates virus-induced fibrosis reactions in rat cardiomyocytes by inhibiting the NF-κB and TGF-β1/Smad2/3 pathways (105). The protective effects of CTRP9 on cardiac injury are mediated by AMPK signaling via AdipoR1 in a mouse model (106). Interestingly, low-dose exercise upregulates CTRP9 expression, and increased cardiac CTRP9 expression contributes to the suppression of heart failure in mice with established myocardial infarction (107). Thus, the upregulation of cardiac CTRP9 via optimized exercise prescriptions may contribute to clinical improvement in patients with CVD. *In vivo*, matrix metalloptoteinase-9 cleaves the secreted full-length CTRP9 into a globular isoform and an N-terminal domain. Notably, Tan et al. demonstrated that the globular isoform of CTRP9 exerts beneficial effects, whereas the N-terminal product promotes cardiac fibrosis by increasing RAP1-MEK/ERK signaling in an *in vivo* myocardial infarction model (108). AdipoR1, the functional receptor for CTRP6 and CTRP9, mediates the suppressive effects of Adiponectin in rat cardiomyocytes (109, 110). Furthermore, AdipoR1 exerts protective effects against fibrosis in neonatal rat atrial myocytes and mouse fibroblasts (89, 111). AdipoR1 is a putative receptor for CTRP7 (29). CTRP7 levels decrease in patients with CAD (70), and CTRP7 contributes to human vascular remodeling *in vitro* (112). Thus, the Adiponectin, CTRP6, CTRP7, and CTRP9/AdipoR1 axes may contribute to the regulation of cardiac fibrosis in CAD and CVD.

White adipose tissue mainly expresses CTRP12; subsequently, furin cleaves full-length CTRP12 to produce a globular isoform. Globular CTRP12 is the major form in the blood (113). Serum CTRP12 levels decrease in patients with CAD (114) and correlate with CAD severity (115, 116). Administration of CTRP12 attenuates cardiac fibrosis and improves cardiac function in an isoproterenol-induced cardiac fibrosis mouse model. This CTRP member inhibits collagen synthesis, α-SMA expression, and cell proliferation in mouse cardiac fibroblasts (117). CTRP12 ameliorates hypoxia/reoxygenation (H/R)- and I/R-induced rat cardiomyocyte injury (118, 119) and prevents cardiac remodeling after myocardial infarction in a mouse model (120). Furthermore, overexpression of CTRP12 alleviates cardiac hypertrophy and fibrosis by modulating the TAK1-p38/JNK pathway, thereby reducing cardiac apoptosis, oxidative stress, and inflammatory responses in post-myocardial infarction heart failure model rats (121). AdipoR2 mediates the suppressive effects of Adiponectin in cardiomyocytes (109, 110). Thus, Adiponectin and the CTRP12/AdipoR2 axes in cardiomyocytes contribute to the regulation of cardiac fibrosis.

CTRP3 levels correlate with disease severity in patients with CAD and CVD (122–125). CTRP3 improves cardiac function by promoting angiogenesis and attenuating cardiac fibrosis by inhibiting myofibroblast differentiation in myocardial infarction mouse and rat model mice (126–130). CTRP3 protects cardiomyocytes against injury via LAMP1; furthermore, CTRP3 replenishment alleviates the deleterious cardiac phenotype in cardiac I/R model mice (130, 131). Thus, LAMP1 may mediate the protective effects of CTRP3 against cardiac disease, while the contribution of the CTRP3/AdipoR2 axis remains unclear. These reports show that CTRPs play an important role in regulating cardiac fibrosis in CAD and CVD via AdipoRs. In fact, AdipoRon ameliorates cardiac necroptosis, apoptosis, cardiac remodeling, and myocardial cell injury in mice (132–135). Thus, AdipoRon has protective effects on heart failure. The adiponectin-derived peptide ALY688, which can bind AdipoR1 and AdipoR2, provides cardioprotection against mouse myocardial ischemic injury (136) and heart failure mouse model (137, 138). Thus, the CTRPs/AdipoRs axes are useful therapeutic targets for cardiac fibrosis.

Researchers have identified Cadherin-13 (also called T-cadherin) as an Adiponectin-binding partner. Adiponectin/Cadherin-13 (T-cadherin) interaction contributes to vascular homeostasis by regulating the transcytosis of circulating Adiponectin to sub-vascular tissues (139, 140). Conversely, soluble T-cadherin ameliorates heart failure and cardiac fibrosis in mice independently of Adiponectin (141), as this soluble cell adhesion molecule does not form a stable complex with Adiponectin from clinical samples (142). AdipoR3 silencing attenuates the inflammatory response and oxidative stress in human glomerular endothelial cells (143) and reduces the expression of adhesion molecules and ECM in human and rat glomerular mesangial cells (144, 145). Thus, this receptor may be involved in the CTRPs-mediated inhibition of cardiac fibrosis.

Serum CTRP1 levels increase in patients with CAD and correlate with disease severity (122, 146, 147). The CTRP1/AMPK pathway inhibits cardiac hypertrophy, fibrosis, oxidative stress, and inflammation in ArgII-induced cardiac hypertrophy model mice (148). Furthermore, CTRP1 protects mouse cardiomyocytes against acute ischemic injury by reducing apoptosis and the inflammatory response (149) and inhibits the collagen-induced platelet aggregation in vascular injury (15). RXFP1, the candidate receptor for CTRP1, contributes to the inhibition of cardiac fibrosis by regulating the endothelial-to-mesenchymal transition in mouse model and human cells (150). Thus, the protective role of CTRP1 on cardiac fibrosis may be mediated via RXFP1. On the other hand, CTRP1 aggravates cardiac fibrosis by promoting collagen transcription in macrophage on post myocardial infarction via AdipoR1 in mouse and rat model (151, 152). Because CTRP1 do not affect rat cardiomyocytes under hypoxic condition, but rather affects the functionality of mouse macrophages (152). Other report showed that CTRP1 promotes PPARγ-dependent pro-atherogenic factors in human macrophages (153). Thus, the role of CTRP1/AdipoR1/PPARγ axis on macrophage may require particular attention in patients with myocardial infarction under hypoxic condition.

Several reports indicate that CTRP5 levels correlate with disease severity in patients with CAD (154–157). CTRP5 attenuates cardiotoxicity in a mouse model by regulating oxidative stress and inflammatory injury in rat myoblasts (158). In contrast, CTRP5 promotes the transcytosis and oxidation of LDL in a mouse CAD model and human aorta endothelial cells lipid uptake model (159), as well as the proliferation, inflammation, and migration of vascular smooth muscle cells, thereby contributing to atherosclerosis progression (154). As with CTRP1, CTRP5 may show cell/tissue-specific pleiotropic effects on CVD. However, overexpression of CTRP5 in the heart attenuates heart injury and failure in a rat model (158, 160), thus suggesting that CTRP5 contributes to positive effects on CVD. These apparently contradictory findings suggest that CTRP1 and CTRP5-mediated signaling are highly context-dependent. Differences in target cell populations, disease stage, metabolic status, and receptor availability may determine whether CTRPs exert protective or pathogenic effects. Future studies using cell type-specific genetic models are required to clarify these context-dependent functions.

Serum CTRP2 levels correlate with disease progression in patients with CAD (161). I/R reduces CTRP2 expression, whereas CTRP2 replenishment improves cardiac function by regulating angiogenesis in mice (162). The role of CTRP4 as a disease marker remains controversial due to conflicting reports on serum CTRP4 levels in patients with CAD (163–165). However, CTRP4 attenuates the development of atherosclerosis in ApoE-deficient mice (165), suggesting that it contributes to the regulation of atherosclerosis. CTRP15 inhibits TGF-β-induced mouse cardiac myofibroblast differentiation, and its overexpression in mouse cardiac myocytes alleviates ventricular remodeling and dysfunction in the heart failure model mice (166). Serum CTRP15 levels increase in patients with CAD and correlate with clinical severity parameters (112, 167). Thus, CTRP15 levels may increase in patients with CAD as a compensatory mechanism to regulate cardiac fibrosis. These cardiovascular protective effects should be distinguished from direct anti-fibrotic effects, as neither angiogenesis nor atherosclerotic plaque formation represents a direct measure of tissue fibrosis.

Previous studies suggest that CTRP5 interacts with AdipoR1, but not AdipoR2 in retinal pigment epithelial (RPE) cells (24). However, the depletion of AdipoR1 and AdipoR2 does not affect the metabolic effects of CTRP5 (25). In addition, CTRP5 interacts with membrane frizzled-related protein (MFRP) in mouse retinal tissue (168). The expression and localization of MFRP in renal tissue remain unknown. Although the functional receptors for CTRP1, CTRP2, CTRP4, CTRP5, and CTRP15 in cardiac tissue remain unidentified, these CTRPs play crucial roles in cardiac fibrosis.

Reports indicate that CTRP11 and CTRP14 contribute to angiogenesis of human endothelial cells via ERK signaling *in vitro* (169). However, CTRP14 is dispensable for atherosclerosis formation in ApoE-deficient mice (170). Clinically, ECM remodeling and fibrosis events are present in abdominal aortic aneurysm (AAA), a life-threatening CAD (171). Plasma CTRP13 levels decrease in patients with AAA; furthermore, CTRP13 replenishment prevents AAA progression by reducing macrophage infiltration and apoptosis of vascular smooth muscle cells in ApoE-deficient mice (172). Endothelial cells contribute to the regulation of cell-matrix adhesion in atherosclerosis. CTRP13 preserves endothelial function in diabetic mice (173). To exert protective effects against atherosclerosis, CTRP13 inhibits HUVEC oxidative stress, apoptosis, and endothelial cell dysfunction (174), and mitigates endothelial cell ferroptosis via the AMPK pathway in HUVECs (175). In fact, CTRP13 overexpression delays the progression of atherosclerosis in ApoE-deficient mice (176). *In vitro* model for myocardial infarction, H/R treatment decreases CTRP13 expression in cardiomyocytes. The CTRP13/AMPK signaling pathway improves cell viability in hypoxia/reoxygenation-subjected cardiomyocytes by inhibiting oxidative stress and apoptosis. CTRP13 protects against myocardial ischemia/reperfusion injury in rats (177). Serum levels of this CTRP member decrease in patients with CAD; thus, CTRP13 serum levels are negative correlative to CAD progression (178). Although the expression and localization of ADGRB3 (BAI3) in cardiac tissue remain unknown, further studies are required to determine whether C1QL family members, including CTRP11, contribute to cardiac fibrosis through ADGRB3.

Collectively, several CTRPs exhibit direct anti-fibrotic effects in cardiovascular models, whereas others exert protective effects through mechanisms that are not directly related to fibrosis, such as angiogenesis, endothelial function, or atherosclerosis. The distinction between these effects is important when interpreting the contribution of individual CTRPs to cardiovascular fibrosis. Although some functional CTRP receptors involved in cardiac fibrosis have been identified, the receptors mediating many of these effects remain unknown. Thus, we the understanding of CTRP-receptor interactions should be enhanced. Despite the encouraging preclinical evidence, most studies rely on gain-of-function approaches or recombinant protein administration in animal models. Genetic loss-of-function studies, cell type-specific knockout models, and validation in large prospective clinical cohorts remain limited. Therefore, the translational relevance of many CTRPs in cardiovascular fibrosis has yet to be fully established. However, an association with the severity of patients with CVD/CAD is evident. These clinical cohorts support the importance of CTRPs in CVD/CAD.

## 4 Roles of CTRPs in the fibrotic responses of non-alcoholic fatty liver disease

Hepatic fibrosis leads to liver dysfunction and has been identified as the most important predictor of prognosis in patients with non-alcoholic fatty liver disease (NAFLD) (179). NAFLD can be described as a metabolic dysfunction including insulin resistance (180). Thus, we may consider the role of CTRPs as adipokine and anti-fibrotic factor (**Figure 1D**).

Clinically, the AdipoR1 gene polymorphism is associated with an increased risk of NAFLD (181), and the AdipoR2 gene polymorphism is associated with the risk of cirrhosis (182). These receptors are expressed in hepatic specimens from obese patients with NAFLD (183). AdipoR1 enhances fatty acid metabolism and cell survival in human hepatocytes (184). In contrast, AdipoR2, but not AdipoR1, exerts anti-fibrotic responses and cell migration in mouse HSCs (185). Furthermore, AdipoR2 overexpression improved nonalcoholic steatohepatitis (NASH) in mice (186).

Serum Adiponectin levels decrease in patients with NAFLD (187, 188), and Adiponectin gene polymorphisms are associated with the risk of NAFLD clinically (189). Replenishment of Adiponectin ameliorates hepatic phenotypes in an NAFLD rat model (190) because Adiponectin improves insulin resistance and other metabolic factors (191). In addition, Adiponectin can regulate the progression of hepatic fibrosis in mouse hepatic stellate cells (HSCs), which is a key driver of local fibrosis (192, 193).

Serum CTRP3 levels decrease in patients with T2DM and NAFLD than in those with T2DM without NAFLD (194). CTRP3 inhibits ECM expression, proliferation, and migration of TGF-β-induced hepatic stellate cells, thereby attenuating hepatic fibrosis in human or mouse cell models (195, 196). As an adipokine, CTRP3 improves insulin sensitivity in mouse preadipocytes (197). Thus, low serum CTRP3 levels may contribute to the prevalence of NAFLD in newly diagnosed patients with T2DM. Thus, Adiponectin and CTRP3 may exert anti-fibrotic effects on liver fibrosis via AdipoR2.

The plasma CTRP5 levels decrease in patients with NAFLD, T2DM, and NAFLD + T2DM, and are negatively correlated with the hepatic fibrosis marker, BMI, and other parameters (198). CTRP5 deficiency attenuates fibrotic and inflammatory states in adipose tissues and metabolic phenotypes in high-fat det-fed mice (199). Furthermore, CTRP5 loss improves insulin action and hepatic steatosis in primary mouse hepatocytes (199). Thus, the blockade of CTRP5 can result in the alleviation of NAFLD. Because CTRP5 interacts with AdipoR1 (24), the CTRP5/AdipoR1 axis may promote the fibrotic response in the liver. AdipoR1 is a putative receptor for CTRP7 in cardiomyoblasts (29). Interestingly, CTRP7 deficiency attenuates liver fibrosis in a high-fat diet mouse model (200). Thus, the CTRP7/AdipoR1 axis may contribute to the exacerbation of hepatic fibrosis (**Figure 1E**).

Adiponectin multimer-mimicked peptides lead to reductions in body weight, blood glucose levels, and hepatic steatosis in a high-fat diet-induced NAFLD mouse model (201). JT003, a dual agonist of AdipoR1 and AdipoR2, reduces hepatic fibrosis in mouse models as well as in both human and mouse cell models (202, 203). Furthermore, AdipoRon shows remedial capacity against hepatic fibrosis in mice (204–206). AdipoR1- and AdipoR2-binding peptide, ADP355 (also known as ALY688), exerts anti-fibrotic and anti-inflammatory effects in mice (207, 208). Studies have also reported conflicting roles of AdipoR1 and AdipoR2 in Th17 cell differentiation from mouse naïve CD4^+^ cells (23, 209). Furthermore, distinct signaling pathways via AdipoR1 and AdipoR2 in the liver support divergent roles of anti-fibrotic ligands (Adiponectin and CTRP3) and pro-fibrotic ligands (CTRP5 and CTRP7) in hepatic fibrosis.

The plasma CTRP1 levels increase in patients with NAFLD, which has a strong association with insulin resistance in NAFLD (210). Because CTRP1 improves insulin sensitivity by enhancing glucose and fatty acid metabolism, the role of CTRP1 in metabolic pathways may contribute to the regulation of NAFLD pathogenesis (211). Serum CTRP4 levels decrease in patients with T2DM and NAFLD than in those without NAFLD (212). Although the role of CTRP4 in hepatic fibrosis is unknown, CTRP4 contributes to the regulation of insulin resistance in mouse preadipocytes (213). As with CTRP3, low serum CTRP4 levels can contribute to the predictor for prevalence of NAFLD with newly diagnosed T2DM. Circulating CTRP9 levels increase in patients with T2DM and are correlated with insulin resistance (214), and CTRP9 attenuates hepatic steatosis in a mouse model (215). However, serum CTRP9 levels are not related to NAFLD in clinical settings (216). Circulating CTRP13 levels also decrease in patients with NAFLD, T2DM, and NAFLD + T2DM, is associated with the increased risk of these diseases, and is negatively correlated with metabolic factors. This study suggests that CTRP13 could be an associated factor with NAFLD in patients with and without T2DM (217). High glucose causes hepatic fibrosis by activating liver sinusoidal endothelial cells (LSECs). CTRP13 can reduce the expression of fibrotic factors in LSECs (218).

Unlike renal and cardiovascular fibrosis, hepatic fibrosis highlights the functional diversity of CTRPs. While Adiponectin and CTRP3 consistently suppress hepatic stellate cell activation and fibrogenesis, CTRP5 and CTRP7 appear to promote metabolic dysfunction and hepatic fibrosis. These findings suggest that the balance among different CTRPs, rather than the activity of a single ligand, may determine the progression of hepatic fibrosis. The opposing effects of CTRPs on hepatic fibrosis may partly reflect receptor selectivity. Anti-fibrotic ligands, including Adiponectin and CTRP3, predominantly signal through AdipoR2 in hepatic stellate cells, whereas pro-fibrotic CTRPs such as CTRP5 and CTRP7 are associated with AdipoR1-mediated metabolic pathways. These observations suggest that receptor specificity may contribute to the functional diversity of CTRPs in the liver.

Hepatic fibrosis develops in close association with systemic metabolic abnormalities. Therefore, the anti-fibrotic effects of several CTRPs may be mediated not only through direct regulation of hepatic stellate cells but also indirectly through improvements in insulin resistance, lipid metabolism, and chronic inflammation. As CTRPs also act as an adipokine, it is necessary to comprehensively understand the role of CTRPs in NALFD.

## 5 Roles of CTRPs in the fibrotic responses of other diseases

CTRP6 alleviates endometrial fibrosis in endometrial stromal cells by regulating the TGF-β/Smad3 pathway in a mouse model of intrauterine adhesions (219). Adiponectin limits the differentiation of human endometrial stromal cells via AdipoR1 and/or AdipoR2 (220). Thus, the loss of CTRP6’s protective role may underlie the abnormal fertility rates observed in CTRP6-deficient mice via the AdipoR1 pathway (26) (**Figure 1F**).

Dermal fibroblasts exhibit myofibroblast phenotypes in lesion skin from patients with vitiligo (221). A genome-wide association study (GWAS) identified the C1QTNF6 gene (encoding CTRP6) as a genetic susceptibility locus significantly associated with generalized vitiligo (222). CTRP6 suppresses TGF-β-induced conversion of fibroblasts to myofibroblasts in human dermal fibroblasts *in vitro* (223, 224). AdipoR1, one of the receptors for CTRP6 (27, 225), is expressed in mouse dermal fibroblasts (226). Thus, the CTRP6/AdipoR1 axis may regulate dermal fibrogenesis, thereby influencing the development of vitiligo.

Systemic autoimmune disease that causes inflammation and irreversible scarring, resulting in fibrosis. Especially, pulmonary fibrosis in rheumatoid arthritis leads to respiratory insufficiency and eventually death (227). Many CTRP mRNA are upregulated in the arthritic joints of rheumatoid arthritis model mice (26). Although the anti-fibrotic role of CTRP3 and CTRP6 in autoimmune disease, CTRP3 contributes to the suppression of rheumatoid arthritis and multiple sclerosis by regulation of Th17 cell differentiation via AdipoR2 in mouse models (23, 228), and CTRP6 controls the development of rheumatoid arthritis as complement regulator (26). The most famous CTRP family, Adiponectin, exerts various biological functions on the development of autoimmune arthritis (229). Adiponectin can inhibit pro-fibrotic activation of human lung fibroblasts as well as in mouse models (230, 231).

Although the role of CTRP9 is unknown, elevated serum CTRP9 levels in patients with systemic sclerosis (SSc) are associated with the fibrotic risk of interstitial lung disease (232, 233). Serum Adiponectin levels decrease in patients with diffuse cutaneous SSc, and low Adiponectin levels are inversely correlated with the cutaneous clinical score (234, 235). To compensate Adiponectin loss, AdipoRon can mitigate cutaneous fibrosis of systemic sclerosis in mice (236).

## 6 Conclusion

This review summarizes current advances in our understanding of CTRP ligands and receptors, as well as their roles in fibrotic responses across various diseases. AdipoRs largely mediate the protective role of CTRPs in fibrosis, although other receptors may also contribute. Many studies have investigated CTRP function using genetically modified mice, RNA interference at single-gene level, or the replenishment of recombinant CTRPs in wild-type mice. Given that CTRPs function in an autocrine manner and can form heteromers (**Table 1**), future studies should investigate the ligand-receptor axes using double- or multiple-gene mutant mice to elucidate the interdependent nature of these molecules. Furthermore, CTRP expression system by linking three monomer sequences together in tandem is helpful for investigating the biological functions of heteromers (237).

Several CTRPs exhibit protective effects against renal, cardiovascular, and hepatic fibrosis, whereas others show context-dependent or even opposing activities. This heterogeneity cannot be explained solely by the expression levels of individual CTRPs. Rather, differences in receptor availability, ligand selectivity, cell type, disease stage, and metabolic or inflammatory context may determine biological outcomes. Importantly, the same CTRP may exert distinct effects in different tissues, suggesting that tissue-specific receptor expression and downstream signaling are critical determinants of CTRP functions. These observations highlight the importance of considering the CTRP network as an integrated regulatory system, rather than evaluating individual CTRPs in isolation.

Regarding the receptors for CTRPs, AdipoRs, BAI, and RXFP1 have been identified. However, the relationships between each CTRP and these receptors have not yet been fully elucidated. As with CTRP2, some CTRPs have not even identified candidate receptors. As Adiponectin receptors, AdipoR1 and AdipoR2 have been identified from the screening of retroviral cDNA expression libraries (11). As receptor candidate for Adiponectin, AdipoR3 has been identified using a yeast-based assay system (13). These methods are useful for receptor identification. As a simple method, RNA interference and peptide blockers can easily identify ligand-receptor interactions (19). To understand the role of CTRPs in fibrosis and other biological functions, we should identify the receptors for each CTRP.

Beyond their metabolic functions as adipokines, CTRPs regulate immune and inflammatory responses, which may contribute to the development and progression of fibrosis. For example, adiponectin and CTRP6 can regulate the complement system (26, 238, 239), whereas adiponectin and CTRP3 can regulate T-cell differentiation from mouse naïve CD4+ T cells (23, 240). CTRP6 has also been implicated in the regulation of inflammatory responses, including those associated with autoimmune and tissue injury. These findings suggest that CTRPs may influence fibrosis not only through direct effects on fibroblasts and extracellular matrix production, but also by modulating the inflammatory and immune environment of injured tissues. Inflammatory and autoimmune diseases can cause repeated or prolonged tissue injury, and persistent inflammation may promote tissue remodeling and fibrosis, ultimately leading to tissue dysfunction (241). Therefore, the immune-regulatory functions of CTRPs should be considered an integral component of CTRP-mediated fibrotic regulation.

Finally, we summarize the future perspective regarding the clinical potential of translation. Because Adiponectin is the most famous CTRP, and is identified as an adipokine (2), a phase 1 clinical trial on AdipoR1/R2 agonist for insulin resistance was reported in 2026 (242). In animal experiments, the AdipoR1/R2 agonist AdipoRon attenuates renal fibrosis (80), intrarenal fibrosis (81), cardiac fibrosis (132–135), and hepatic fibrosis (204–206) in mice. The AdipoR1/R2-binding peptide ALY688 modulates cardiac fibrosis (136–138) and hepatic fibrosis (207, 208). These results suggest that the CTRP/AdipoR axis is an useful target for fibrotic diseases. Following promising research results, the initiation of clinical trials targeting fibrotic diseases is anticipated.
